# A systems-level integration of liver-kidney transcriptomics and genome-scale metabolic models reveals organ-specific injury mechanisms

**DOI:** 10.3389/ftox.2026.1866643

**Published:** 2026-07-06

**Authors:** Venkat R. Pannala, Archana Hari, Scott S. Auerbach, Anders Wallqvist

**Affiliations:** 1 Department of War Biotechnology High Performance Computing Software Applications Institute, Defense Health Agency Research & Development, Medical Research and Development Command, Fort Detrick, MD, United States; 2 The Henry M. Jackson Foundation for the Advancement of Military Medicine, Inc., Bethesda, MD, United States; 3 Division of Translational Toxicology, National Institute of Environmental Health Sciences, Research TrianglePark, NC, United States

**Keywords:** bile acid export, cell cycle regulation, ER stress, lipid metabolism, liver and kidney toxicity, transcriptomics

## Abstract

The liver and kidneys are the primary organs that clear chemicals from the body, yet they often exhibit different pathological responses to the same systemic exposure. While high-throughput transcriptomics (HTT) can map broad molecular perturbations, identifying the differential mechanisms that govern organ-specific injury remains a challenge. To address this knowledge gap, we investigated the dose-dependent effects of three peroxisome proliferator-activated receptor alpha agonists—coumarin, fenofibrate, and perfluorooctanoic acid (PFOA)—on liver and kidney metabolism to understand the organ-specific mechanisms of toxicity. We used HTT data from 5-day *in vivo* rat exposure studies and performed a comparative analysis on the paired liver and kidney data, using gene co-expression and pathway enrichment analyses together with genome-scale models, to investigate their differential sensitivity to chemical exposure. Our results revealed that all three chemicals caused larger gene perturbations in the liver compared to the kidney, in agreement with their well-known hepatotoxicity. Fenofibrate and PFOA triggered a profound upregulation in pathways related to fatty acid metabolism but suppressed several pathways related to amino acid metabolism in both organs. All three chemicals showed a strong upregulated antioxidant response in the liver, indicating an adaptive response to chemical stress, with fenofibrate and PFOA entering uncontrolled endoplasmic reticulum stress, which was also observed for PFOA in the kidney. Our comparative analysis revealed a striking divergence in cellular repair mechanisms in response to stress across both tissues: while the liver strongly upregulated apoptotic and cell-cycle repair pathways, signaling active hepatotoxicity, the kidney consistently downregulated these same vital processes in response to all three chemicals, indicating differential mechanisms that may lead to organ toxicity.

## 1 Introduction

The liver and kidneys serve as the primary organs for the metabolism, distribution, and clearance of xenobiotics, making them highly susceptible to chemical-induced injury (Vree et al., 1992; Pizzo et al., 2015; Almazroo et al., 2017). Understanding the molecular mechanisms underlying these organ-specific injuries is a critical challenge in toxicological risk assessment. In recent years, high-throughput transcriptomics (HTT) has emerged as a powerful new approach methodology to decipher the complex mechanisms of chemical toxicity and identify sensitive molecular points of departure (Farmahin et al., 2017; Gwinn et al., 2020; Alcaraz et al., 2022; Reardon et al., 2023). By evaluating a system-wide characterization of gene expression, transcriptomics allows researchers to effectively identify perturbed molecular pathways, map cellular stress responses, and capture a large biological response space that precedes overt apical toxicity. Integrating transcriptomic profiling across these paired “sentinel” tissues can reveal the mechanisms behind toxic chemical exposures, which serve as early indicators of potential toxicological hazards, facilitating timely and cost-effective screening of xenobiotic-mediated stress responses.

To rigorously evaluate toxicological mechanisms, the selection of chemicals that share common molecular targets but exhibit divergent adverse outcomes provides a unique analytical advantage. The peroxisome proliferator-activated receptor alpha (PPARα) is a ligand-activated nuclear transcription factor that acts as a master regulator of lipid metabolism, fatty acid oxidation, and energy homeostasis, and it is predominantly expressed in tissues with high metabolic rates, such as the liver and kidneys (Mandard et al., 2004; Bookout et al., 2006; Varga et al., 2011). A diverse array of chemicals are known to activate PPAR nuclear receptors, including pervasive environmental contaminants [e.g., perfluorooctanoic acid (PFOA)] (Louisse et al., 2023; Golden-Mason et al., 2025), targeted synthetic pharmaceuticals (e.g., fenofibrate) (Lakhia et al., 2018; Zhou et al., 2026), and naturally occurring plant derivatives (e.g., coumarins) (Um et al., 2013; Rigano et al., 2017). Despite sharing PPARα agonism, these chemicals produce vastly different toxicity profiles. For instance, PFOA and fenofibrate can induce massive hepatic lipid overdrive, cellular hypertrophy, and steatosis (Yamamoto et al., 1996; Zubrzycki et al., 2020; Louisse et al., 2023). Both chemicals also demonstrate significant kidney toxicity, with PFOA exposure associated with altered urea metabolism and renal damage, and fenofibrate linked to multiple cellular stress pathways in the kidney (Liu et al., 2022; Wronska et al., 2024; Wu et al., 2025). Conversely, high-dose coumarin exposure is classically linked to oxidative stress, generic tissue necrosis, and hepatotoxicity, with reported occurrences of nephropathy and renal tubular adenomas (Uehara et al., 2008; Gwinn et al., 2020; Pitaro et al., 2022). Therefore, to connect these pathophysiological changes to specific organ liabilities, which will reveal how shared receptor activation or individual differences can diverge into distinct, tissue-specific pathological trajectories, we need integrated transcriptomic studies utilizing multi-organ, network-level comparisons.

Although there are several analytical methods available to evaluate transcriptomic data, significant gaps remain in providing comprehensive systems-level interpretations. For example, traditional pathway enrichment methods can only evaluate static lists of differentially expressed genes to identify affected biological processes and associated modules for injury (Tawa et al., 2014; Alcaraz et al., 2022; Pannala et al., 2023). These conventional methods fail to account for the complex interactions of multiple genes catalyzing interconnected metabolic reactions and struggle to resolve directional metabolic consequences. Therefore, they often cannot identify the specific functional bottlenecks that link transcriptomic stress responses directly to phenotypic toxicity. Genome-scale metabolic models (GSMs) offer a robust systems-level solution to this limitation by mathematically representing the entire metabolic network of an organism (Blais et al., 2017; Pannala et al., 2025b). By integrating transcriptomic data into GSMs using constraint-based algorithms, researchers can computationally predict quantitative metabolic flux distributions (the fluxome), thereby translating static gene-expression changes into dynamic, directional metabolic alterations and uncovering critical mechanistic bottlenecks driving toxicity (Pannala et al., 2020; Hari et al., 2025).

In this study, we combined traditional transcriptomic analysis methods with advanced GSMs to elucidate the mechanistic differences in target-organ responses to chemical exposures. We utilized HTT data from male rats exposed to varying doses of three distinct PPARα agonists: coumarin, fenofibrate, and PFOA (Gwinn et al., 2020). By analyzing paired liver and kidney transcriptomes, we aimed to provide an enhanced, comprehensive picture of organ-specific toxicity. To this end, we explored the data through two complementary analytical lenses: first, we used traditional transcriptomic methods, including dose-dependent hierarchical clustering and comparative pathway-based enrichment mapping, to evaluate the sensitivity of perturbed molecular pathways, identify points of departure, and trace localized cellular response mechanisms, such as xenobiotic metabolism, lipid regulation, cell cycle regulation, and apoptosis. Second, we employed an additional GSM analysis to showcase the dynamic, directional metabolic aspects of liver and kidney metabolism, highlighting shifts in lipid, amino acid, and bile acid regulation. By integrating traditional pathway analyses with GSM flux predictions, this study offers a robust methodology to comprehensively map the divergent metabolic trajectories and distinct cellular survival strategies that dictate differential apical injury outcomes in the liver and kidneys.

## 2 Materials and methods

### 2.1 Animal use, chemical exposures, and tissue collection

We obtained all of the experimental data used in the current study from the previously published study of Gwinn et al. (2020). In their work, Gwinn et al. (2020) provided a detailed description of the entire study protocol used for the 5-day *in vivo* rat studies. Briefly, all the *in vivo* studies were conducted in facilities accredited for laboratory animal care and were reviewed and approved by the Battelle Animal Care and Use Committee. The study adhered to the National Institutes of Health Guide for the Care and Use of Laboratory Animals and complied with institutional and federal requirements for humane handling, housing, and euthanasia. Male Sprague Dawley (Hsd: Sprague Dawley SD) rats were obtained from an approved vendor (Envigo, Haslett, MI, United States) and were acclimated for ∼2 weeks under controlled temperature and humidity with a 12-h light/dark cycle. Animals were housed in individually ventilated cages and had access to an irradiated NTP-2000 diet and water *ad libitum*. At the start of the study, animals were between 8 and 10 weeks of age and were randomized into exposure groups using body-weight stratification to minimize baseline variation. The rats were evaluated twice daily over the course of the study for moribundity and mortality.

The selected chemicals (e.g., coumarin, PFOA, and fenofibrate) were administered once daily for five consecutive days by oral gavage at dose levels selected to encompass and extend previously characterized toxicological ranges (National Toxicology Program, 1993; Gwinn et al., 2020). The study contained seven dose levels for coumarin (3.125, 6.25, 12.5, 25, 50, 100, 200 mg/kg) with corn oil as the control group, eight dose levels for PFOA (0.156, 0.3125, 0.625, 1.25, 2.5, 5, 10, 20 mg/kg) with 2% Tween80 as the control, and eight dose levels for fenofibrate (8, 16, 31.25, 62.50, 125, 250, 500, 1,000 mg/kg) with 0.5% aqueous methylcellulose solution as the control (n = 4 rats per condition). Twenty-four hours after the final gavage, animals were anesthetized using a controlled 70% CO_2_:30% O_2_ gas mixture and euthanized by exsanguination, as described in the original study (Gwinn et al., 2020). The liver and both kidneys were removed, weighed, and examined visually. The left liver lobe and right kidney were isolated for transcriptomic analysis, placed in RNAlater stabilization solution, and held overnight at 4 °C to ensure thorough reagent penetration. Once the RNAlater supernatant was removed, they were stored at low temperature until RNA extraction.

### 2.2 RNA isolation and sequencing

Total RNA was isolated from liver and kidney samples using RNeasy Mini Kit (Qiagen) that included an on-column DNase digestion step to eliminate genomic DNA contamination. RNA integrity was confirmed by electrophoretic assessment, and absorbance ratios (260/280) were used to verify purity prior to library preparation. HTT profiling was performed using the rat S1500^+^ TempO-Seq platform, a hybridization-based targeted sequencing assay designed to capture ∼3,000 transcripts representing over 85% of curated biological pathways (Mav et al., 2018). The method involved hybridization of mRNA molecules with paired detector oligonucleotides, nuclease removal of unbound probes, incorporation of sample barcodes through polymerase chain reaction, and pooling of amplicons for downstream sequencing.

Sequencing was performed on an Illumina HiSeq 2,500 Ultra High-Throughput Sequencing System (Illumina, San Deigo, CA, United States), generating FASTQ files for each biological replicate. Reads were aligned to the S1500^+^ probe set using Bowtie version 1.2.2 (Langmead et al., 2009), with settings permitting up to three mismatches and reporting only the single best alignment for each read. Sample quality was evaluated using criteria established for TempO-Seq data processing, including thresholds for total sequencing depth, alignment rate, proportion of probes reaching detectable coverage, and total aligned reads. Samples with fewer than 500,000 total reads, alignment rates below 40%, unique alignment rates below 30%, percent probes with at least five reads below 50%, and number of aligned reads fewer than 500,000 were excluded from downstream analysis. Additional exclusions were made when principal component analysis (PCA), hierarchical clustering, or inter-replicate correlations indicated outlier behavior (Gwinn et al., 2020). Following alignment, raw counts were processed according to the TempO-Seq attenuation model to estimate unattenuated transcript abundance, and normalized counts were generated using a counts-per-million framework to account for sequencing depth variability. A pseudocount of 1.0 was added to all features before log_2_ transformation.

### 2.3 Differential gene expression and pathway enrichment analysis

Given the log_2_ transformed experimental data, in this study we performed differential gene expression analyses independently for the liver and kidney. We applied a one-way analysis of variance (ANOVA) across all dose groups to identify dose-dependent transcriptional responses, followed by multiple-comparison testing using the least significant difference approach (anovan function in MATLAB). To control for false positives, we calculated adjusted p-values using the Benjamini–Hochberg false discovery rate (FDR) procedure (mafdr function in MATLAB). We considered genes significantly altered when they met both the FDR threshold (<0.1) and a minimum absolute log_2_ fold change (>0.3). We used the resulting differentially expressed gene (DEG) fold-change values for all our subsequent analyses. We conducted organ-specific biological interpretation of DEGs using KEGG pathway enrichment analyses based on an aggregated fold-change (AFC) strategy (Yu et al., 2017; Schyman et al., 2021), which quantified pathway-level upregulation or downregulation by integrating the magnitude and directionality of gene-level responses. For this analysis, we used significantly differentially expressed genes together with their fold-change values at the highest dose for each chemical with FDR <0.1 and log_2_ fold change value > |0.3| compared to their respective control groups as input. In addition, to overcome the database-based bias in our pathway analysis, we also performed pathway enrichment analysis of curated Reactome and WIKI pathways using ShinyGO, an interactive platform that supports hierarchical gene-set clustering and identification of functional relationships among perturbed pathways (Ge et al., 2020). We selected the rat genome version mRatBN7.2 as the background gene set and identified the top 30 significantly altered pathways based on FDR (<0.001) and sorting by fold enrichment. For this analysis, we used the same gene selection criteria as explained for the KEGG pathway analysis, however, we used only gene symbols as input. To generate the dose-response plots for each major pathway, we identified significantly enriched subpathways (FDR <0.05) from the results of all three pathway enrichment analysis platforms, combined all the genes from those pathways, and identified unique genes for that major pathway.

### 2.4 Genome-scale metabolic modeling of transcriptomic data

To characterize metabolic pathway alterations at the systems level, we integrated the transcriptomic datasets with a rat GSM comprised of more than 13,000 reactions distributed across over 50 metabolic subsystems (Hari et al., 2025; Pannala et al., 2025b). We previously provided a detailed description of the methodology used to generate gene expression-based flux distributions for each condition and organ (Hari et al., 2025). Briefly, we mapped gene expression measurements from the S1500^+^ dataset to metabolic reactions using established gene–protein–reaction association rules. Overall, the rat GSM contains ∼3,102 unique genes, and our experimental data covers approximately 21% of them from each studied chemical. We performed flux estimation using the Pheflux algorithm (Gonzalez-Arrue et al., 2023), which applied an entropy-based optimization framework to identify flux distributions most consistent with observed transcript abundance while maintaining stoichiometric mass balance. For each animal, reaction-level fluxes were aggregated to subsystem-level activity scores, allowing quantification of dose-dependent metabolic perturbations. We compared the subsystem flux responses between the liver and kidney using hierarchical clustering and PCA. All the methods used to present the results in this study were independently assessed for reproducibility.

## 3 Results

### 3.1 Effects of 5-day chemical exposure on rat body and organ weights

**FIGURE 1:**
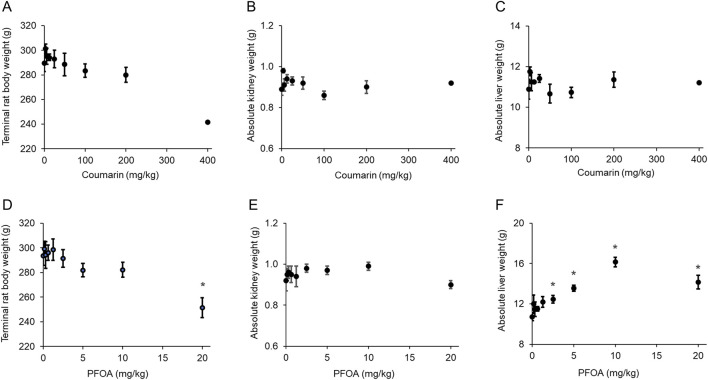
Effect of 5-day chemical exposure on rat body and organ weights. Alterations in rat terminal body weight: **(A)** coumarin and **(D)** perfluorooctanoic acid (PFOA). Alterations in absolute kidney weight: **(B)** coumarin and **(E)** PFOA. Alterations in absolute liver weight: **(C)** coumarin and **(F)** PFOA. *Statistically significant difference between treatment and vehicle control groups (p-value <0.01). The experimental raw data for the rat body weights and organ weights were obtained from the Supplementary Database files of the original Gwinn et al. (2020) study.

We used a 5-day *in vivo* rat exposure study to assess the physiological and organ-specific impacts of coumarin and PFOA on rat terminal body, absolute liver, and kidney weights (Figure 1). Following coumarin administration across a dose range of 0–400 mg/kg, absolute kidney and liver weights remained stable, exhibiting no significant changes at any dose level (Figures 1B,C). However, we observed systemic effects at the highest concentration, with a marked reduction in terminal body weight at the maximum dose (400 mg/kg) (Figure 1A). In contrast, the 5-day exposure to PFOA (0–20 mg/kg) induced a robust, dose-dependent increase in absolute liver weight. These statistically significant hepatic elevations occurred even at the lower doses and remained through the peak dose (20 mg/kg) (Figure 1F). Consistent with the coumarin study, PFOA administration did not significantly alter absolute kidney weights (Figure 1E). Furthermore, PFOA exposure resulted in a statistically significant decline in terminal body weight at the highest tested dose (20 mg/kg) (Figure 1D), indicating that both chemicals induced high-dose systemic effects but only PFOA caused a pronounced toxic response. We previously reported a similar trend in absolute rat liver weights for fenofibrate exposure (Pannala et al., 2025a), suggesting potential similar mechanisms involved in the development of liver hypertrophy.

### 3.2 Coumarin induced dose-dependent alterations in rat liver and kidney metabolism

**FIGURE 2:**
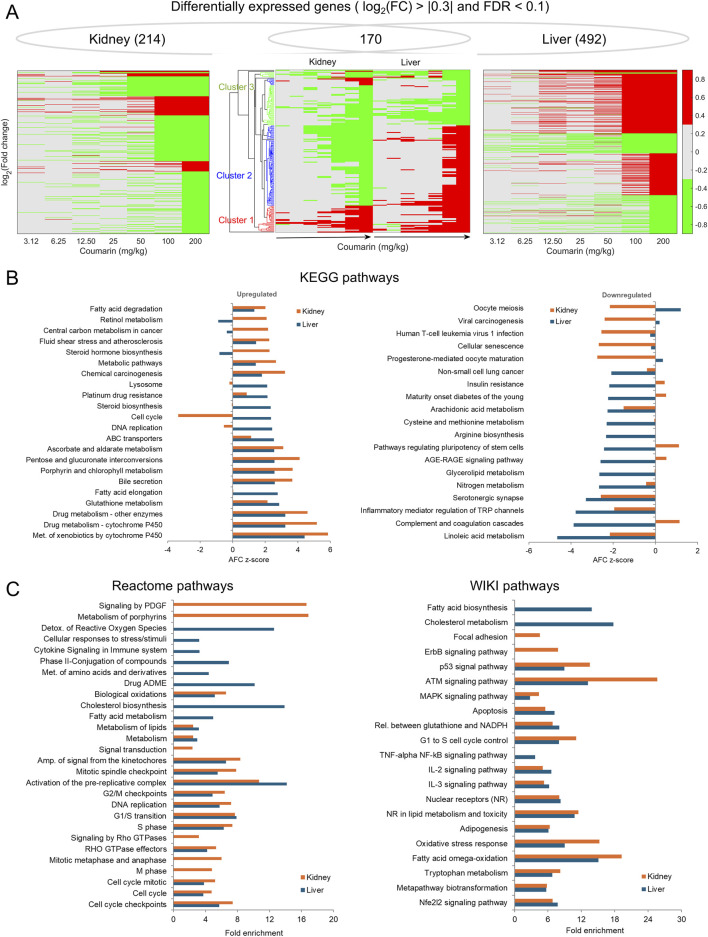
A transcriptomic and pathway-level alteration map for the kidney and liver in response to coumarin exposure. **(A)** Heatmap of significantly perturbed genes in the rat kidney (left panel) and liver (right panel) and the genes common between them (center panel) in response to increased coumarin doses. The total number of significantly perturbed genes (indicated in parentheses) for each tissue was obtained based on a false discovery rate <0.1 and log2 fold-change (FC) values > |0.3|. **(B,C)** Significantly perturbed biological pathways in the kidney and liver at the highest concentration based on KEGG **(B)** as well as Reactome and WIKI **(C)** pathway enrichment analysis. AFC, aggregated fold change.

To evaluate the molecular mechanisms driving the physiological responses to coumarin following the 5-day *in vivo* exposure, we performed HTT profiling and one-way ANOVA on the rat liver and kidney tissues. Figure 2A shows a manual clustering-based heatmap to visualize the data for the kidney (left panel) and the liver (right panel) based on the gene fold-change and significance threshold value cut offs of log2(FC) > |0.3| and FDR <0.1, respectively. Our results revealed distinct, dose-dependent transcriptomic alterations that became pronounced at the highest coumarin concentrations (100 and 200 mg/kg) for both the kidney and liver (Figure 2A). Coumarin exposure induced a more robust overall transcriptional response in the liver compared to the kidney; specifically, we identified 492 unique DEGs in the liver, with the majority of them upregulated (red), compared to 214 unique DEGs in the kidney, with most of them downregulated (green) at the highest dose. Furthermore, coumarin exposure commonly altered an overlapping set of 170 DEGs in both organs (Figure 2A, center panel). Hierarchical clustering of these shared genes (denoted as Clusters 1, 2, and 3) demonstrated that while both tissues co-regulated certain gene sets in the same direction, other gene sets exhibited divergent, tissue-specific expression patterns in response to coumarin toxicity.

To elucidate the functional impact of these transcriptomic shifts, we conducted pathway enrichment analyses using the ToxPanel (Schyman et al., 2021) and ShinyGo (Ge et al., 2020) platforms across the KEGG, curated Reactome, and curated WIKI databases (Figures 2B,C). In both the liver and kidney, KEGG analysis demonstrated a robust upregulation of pathways critical for chemical clearance and cellular defense, notably drug metabolism via cytochrome P450 and glutathione metabolism, alongside lipid processing pathways, such as fatty acid degradation (Figure 2B). Conversely, coumarin exposure predominantly downregulated several pathways related to immune response, signaling, and cellular homeostasis, including linoleic acid metabolism and inflammatory mediator regulation. Notably, KEGG pathway analysis also revealed a striking, tissue-specific divergence regarding cell proliferation and genomic maintenance. For example, while pathways governing the cell cycle and DNA replication were upregulated in the liver (exhibiting positive AFC scores), these exact same processes were distinctly downregulated in the kidney (Figure 2B). Indeed, Reactome pathway analysis corroborated these biological classifications, highlighting significant fold enrichments in several cell cycle regulation-related processes (e.g., G_1_/S transition, G_2_/M checkpoints, S phase) in both the liver and kidney as well as alterations in biological oxidations, phase II conjugation of compounds, and detoxification of reactive oxygen species (ROS) (Figure 2C). Furthermore, WIKI pathway profiling revealed significant enrichment in Nfe2l2 signaling, oxidative stress responses, and nuclear receptor (NR)-mediated lipid metabolism and toxicity. Collectively, these results indicate that coumarin exposure triggered a massive, dose-dependent xenobiotic detoxification and oxidative stress response, which simultaneously induced significant reprogramming of cellular lipid and fatty acid metabolism in both hepatic and renal tissues, and had a highly divergent, organ-specific impact on cell cycle progression and DNA replication trajectories.

#### 3.2.1 Dose-response behavior of key perturbed pathways in response to coumarin exposure in the liver and kidney

**FIGURE 3:**
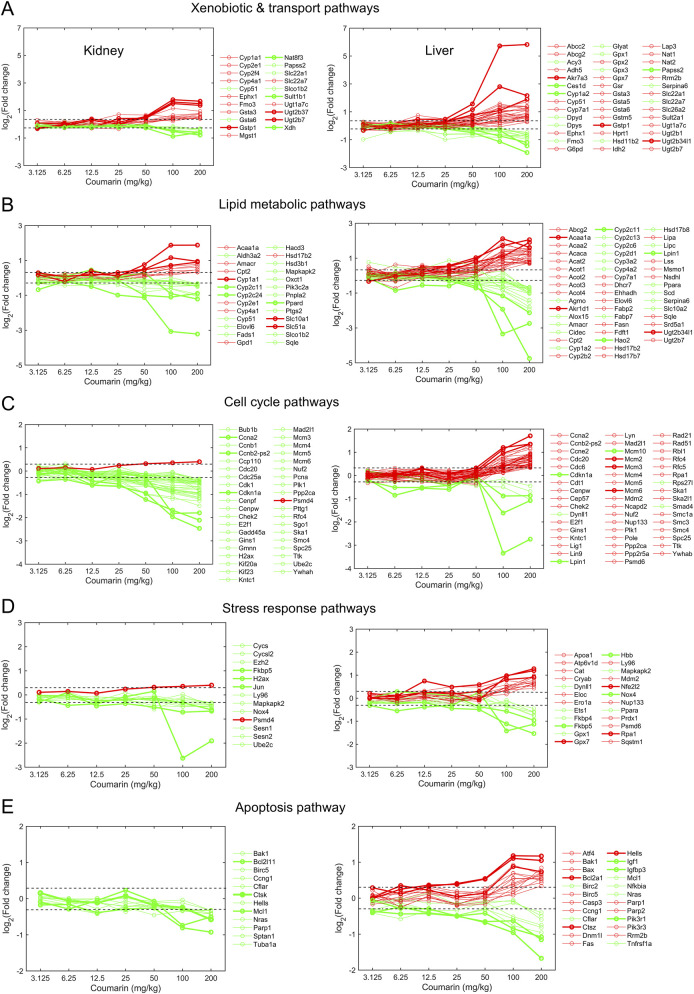
Pathway-based dose-response profiles for significantly perturbed genes in the kidney and liver in response to coumarin exposure. Line plots show the logarithmic fold-change (FC) values for genes significantly altered (false discovery rate <0.1) by coumarin in the **(A)** xenobiotic and transport pathways, **(B)** lipid metabolism, **(C)** cell cycle, **(D)** stress response, and **(E)** apoptosis pathways. Dashed lines show genes that satisfy the log2FC > |0.3| criterion at the highest coumarin dose.

To further characterize the organ-specific molecular mechanisms driving coumarin toxicity, we analyzed the individual dose-response trajectories of genes within the most significantly enriched biological pathways (Figure 3). Consistent with the global pathway data, the individual gene profiles demonstrated that the liver had a substantially higher-magnitude transcriptional response than the kidney. Within the xenobiotic metabolism (Figure 3A) and lipid metabolic pathways (Figure 3B), we observed a massive, dose-dependent upregulation (indicated by red lines) of numerous genes in the liver—including members of the cytochrome P450 (*Cyp*) family, glutathione S-transferases (*Gst*), and various lipid catabolism enzymes—which surged abruptly at the highest concentrations (100 and 200 mg/kg). While the kidney also upregulated a subset of these clearance and metabolic genes, the overall magnitude of the fold change and the total number of recruited genes were markedly lower, with the kidney simultaneously downregulating (indicated by green lines) several specific lipid metabolism genes that the liver actively upregulated.

Importantly, individual gene expression profiling confirmed the striking organ-specific divergence in cell proliferation and genomic maintenance (Figure 3C). In the liver, high-dose coumarin exposure triggered a robust upregulation (red) of critical cell cycle and DNA replication drivers, including key cyclins (e.g., *Ccna2*, *Ccnb2*) and minichromosome maintenance (*Mcm*) complex genes, strongly suggesting a compensatory proliferative or regenerative response to hepatic injury. Conversely, the kidney profoundly downregulated (green) these exact same cell cycle genes across the dosage spectrum, pointing to a definitive cell cycle arrest or a suppression of normal renal cellular proliferation. Furthermore, this divergent cellular trajectory was mirrored in the stress response (Figure 3D) and apoptosis pathways (Figure 3E). As coumarin doses reached 100 and 200 mg/kg, the liver exhibited a distinct upregulation of numerous stress and apoptotic genes, signaling the onset of severe cellular damage and the activation of programmed cell death mechanisms (e.g., *Bak1*, *Bax*, *Casp3*, *Ctsz*). In stark contrast, the kidney predominantly downregulated or maintained baseline expression across these same stress and apoptotic gene networks. Overall, these gene-level dose-response profiles demonstrate that while both organs initiated protective xenobiotic clearance and lipid reprogramming, high-dose coumarin exposure specifically drove the liver toward severe oxidative stress, apoptosis, and compensatory proliferation, whereas the kidney responded by avoiding apoptosis and prioritizing metabolic suppression and cell cycle arrest.

### 3.3 PFOA induced dose-dependent alterations in rat liver and kidney metabolism

**FIGURE 4:**
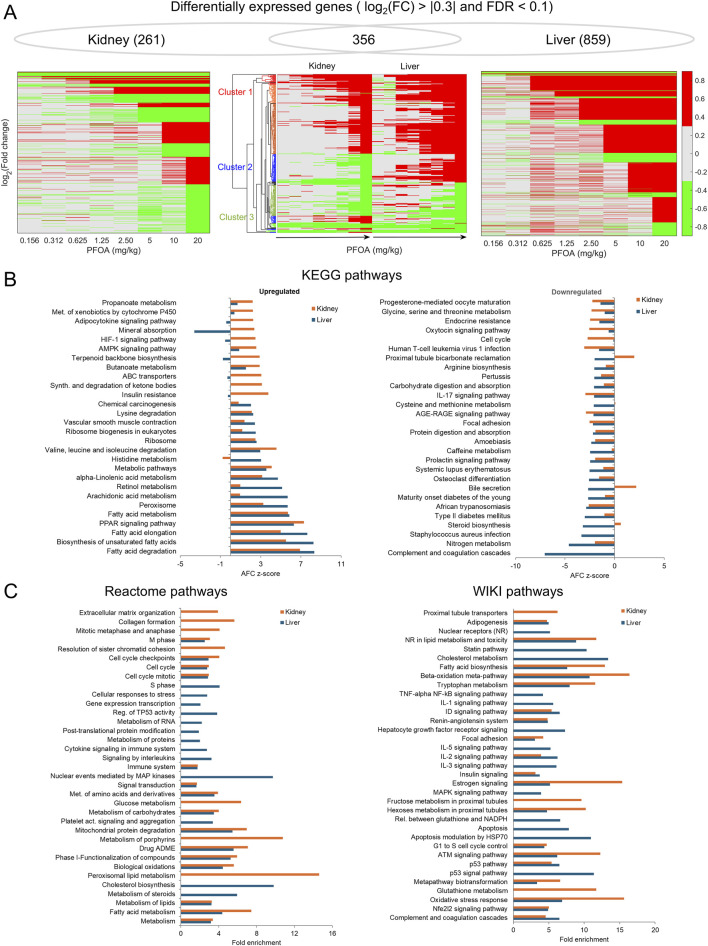
A transcriptomic and pathway-level alteration map for the kidney and liver in response to perfluorooctanoic acid (PFOA) exposure. **(A)** Heatmap of significantly perturbed genes in the rat kidney (left panel) and liver (right panel) and the genes common between them (center panel) in response to increased PFOA doses. The total number of significantly perturbed genes (indicated in parentheses) for each tissue was obtained based on a false discovery rate <0.1 and log2 fold-change (FC) values > |0.3|. **(B,C)** Significantly perturbed biological pathways in the kidney and liver at the highest concentration based on KEGG **(B)** as well as Reactome and WIKI **(C)** pathway enrichment analysis. AFC, aggregated fold change.

Following the same gene significance criteria defined for the coumarin study [log2(FC) > |0.3| and FDR <0.1], we evaluated the molecular mechanisms driving the systemic and organ-specific responses to PFOA in the kidney and liver. Figure 4A shows a manual clustering-based heatmap to visualize the dose-dependent transcriptomic alterations for the kidney (left panel) and liver (right panel) and unsupervised hierarchical clustering for genes common between them (center panel). Consistent with our previous analyses, red indicates significantly upregulated genes, representing the activation of adaptive metabolic reprogramming and detoxification mechanisms, while green indicates significantly downregulated genes, reflecting the suppression of baseline cellular homeostasis. PFOA induced a massive, dose-dependent transcriptional disruption that became pronounced at the 5, 10, and 20 mg/kg concentrations. The liver exhibited a drastically higher sensitivity and magnitude of response (859) compared to the kidney (261) with respect to unique genes, however, there were several commonalities in their directionality among the common genes (356 DEGs), as indicated by Clusters 1 and 3.

Figures 4B,C show the pathway enrichment analyses, based on individual significantly altered genes for the liver and kidney as inputs, that elucidate the functional biological impact of these alterations. In both the liver and the kidney, the highly upregulated (red) gene clusters mapped predominantly to a massive reprogramming of cellular lipid metabolism. KEGG and Reactome analyses revealed robust, dose-dependent enrichments in the PPAR signaling pathway, peroxisome activity, fatty acid degradation, and biological oxidations (Figures 4B,C). This massive upregulation aligns with the known mechanism of highly fluorinated compounds acting as potent PPAR agonists (Zhang et al., 2014; Pederick et al., 2024). Conversely, we observed that the downregulated (green) gene clusters in both organs mapped to the suppression of focal adhesion, nitrogen metabolism, and IL-17 signaling pathways. However, cell cycle regulation was predominantly downregulated in the kidney, whereas bile secretion was upregulated (Figures 4B). Furthermore, based on WIKI pathway analyses, we observed a strong perturbation in cholesterol metabolism only in the liver and glutathione metabolism only in the kidney (Figure 4C). Collectively, these results indicate that PFOA exposure induced a massive, PPAR-mediated reprogramming of lipid and peroxisomal metabolism and an accompanied oxidative stress response across both hepatic and renal tissues.

#### 3.3.1 Gene-level dose-response dynamics within key perturbed pathways following PFOA exposure

**FIGURE 5:**
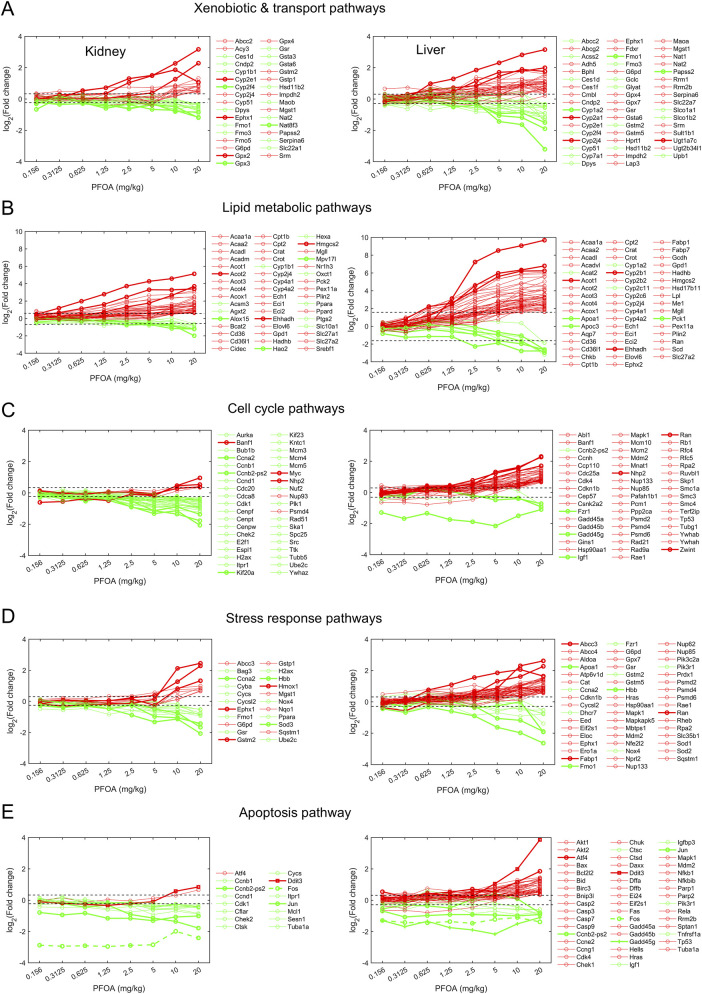
Pathway-based dose-response profiles for significantly perturbed genes in the kidney and liver in response to perfluorooctanoic acid (PFOA) exposure. Line plots show the logarithmic fold-change (FC) values for genes significantly altered (false discovery rate <0.1) by PFOA in the **(A)** xenobiotic and transport pathways, **(B)** lipid metabolism, **(C)** cell cycle, **(D)** stress response, and **(E)** apoptosis pathways. Dashed lines show genes that satisfy the log2FC > |0.3| criterion at the highest dose of PFOA for all the figures except **(B)**. In **(B)**, the dashed line indicates the log2FC > |0.6| and > |1.6| for the left and right panels, respectively.

To further dissect the organ-specific molecular mechanisms driving the physiological response to PFOA, we analyzed the individual dose-response trajectories of genes within the most significantly enriched biological pathways (Figure 5). Acting as a potent PPARα agonist, PFOA induced a massive, dose-dependent upregulation (indicated by red lines) of xenobiotic and lipid metabolic pathways, predominantly functioning as an adaptive cellular response in the liver. Within the xenobiotic metabolism network (Figure 5A), we observed a robust induction of phase I and phase II clearance enzymes; specifically, the genes *Cyp2e1*, *Cyp2j4*, *Ephx1*, and *Cyp51* surged rapidly both in the kidney (left panel) and the liver (right panel) at higher concentrations. Concurrently, PFOA exposure triggered a massive transcriptional reprogramming of lipid metabolic pathways (Figure 5B) in both organs, though the magnitude was drastically higher in the liver, with many gene log2FC values perturbed by more than 1.6 (Figure 5B, right panel). This adaptive metabolic shift was characterized by an aggressive upregulation of genes governing peroxisomal and mitochondrial fatty acid oxidation, most notably *Acox1*, *Acot1*, *Ehhadh*, *Cpt1b*, *Cd36*, and *Acaa1a*, which steadily increased in expression across the dosage spectrum.

Importantly, individual gene expression profiling of the cell cycle pathways (Figure 5C) confirmed highly divergent cellular growth trajectories between the two organs. In the kidney, PFOA profoundly suppressed normal cellular division, as demonstrated by the widespread, dose-dependent downregulation (indicated by green lines, reflecting the suppression of baseline homeostasis) of critical cell cycle drivers, kinases, and mitotic regulators, including *Ccnb1*, *Cdk1*, and *Cdc20*. In stark contrast, the liver exhibited a highly active, predominantly upregulated response to PFOA exposure. While a few specific regulators were downregulated (e.g., *Fzr1*, *Ccnb2*, *Gadd45g*), the liver robustly upregulated a large cluster of cell cycle and structural maintenance genes, including *Mcm10*, *Ran*, *Zwint*, and *Rb1*, as the dose increased. This indicates that active cell cycle signaling and compensatory proliferation occurred alongside the massive metabolic hypertrophy in the liver.

Finally, we evaluated the gene-level dynamics within the stress response (Figure 5D) and apoptosis pathways (Figure 5E). To counteract the massive metabolic shifts induced by PFOA at the 5-, 10-, and 20-mg/kg doses, the liver mounted a pronounced antioxidant and cellular stress response, as demonstrated by the dose-dependent upregulation (red) of key oxidative stress and detoxification genes (e.g., *Nfe2l2*, *Cat*, *Gpx7*, *Gsr*, *Sod1*, and *Sod2*), indicating activation of the NRF2 pathway in response to cellular stress (Figure 5D, right panel). We observed upregulation of several stress response genes, including *Hmox1*, *Nqo1*, *G6pd*, *Mgst1*, and *Gstm2*, indicating activation of NRF2 pathway also in the kidney (Figure 5D, left panel). Furthermore, the liver upregulated specific endoplasmic reticulum (ER) stress and apoptotic mediators, i.e., *Atf4*, *Bax*, *Ddit3*, and several Casp genes, signaling the onset of severe cellular damage and lipotoxicity at peak exposure levels. In contrast, except for the genes related to ER stress (*Atf4* and *Ddit3*), the kidney predominantly downregulated (green) or maintained baseline expression across these exact same stress and apoptotic networks, prioritizing metabolic suppression of genes in programmed cell death. These individual gene profiles confirm that while PFOA systematically suppressed the cell cycle in the kidney, it simultaneously forced the liver into a complex state of hypertrophic metabolic disruption, active cell cycle signaling, and severe oxidative stress.

### 3.4 Fenofibrate induced dose-dependent alterations in rat liver and kidney metabolism

**FIGURE 6:**
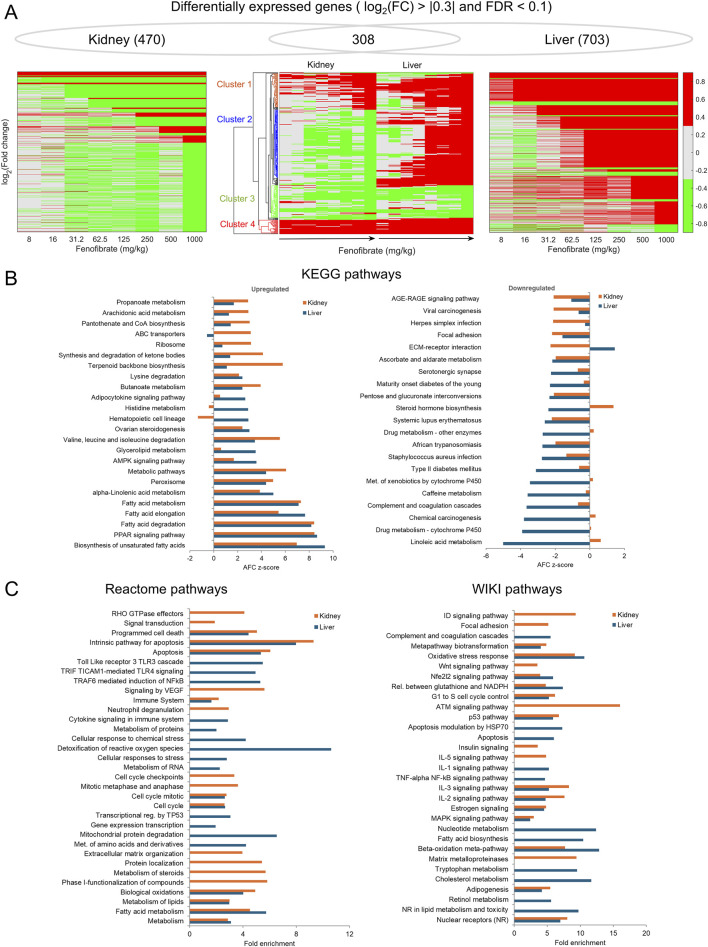
A transcriptomic and pathway-level alteration map for the kidney and liver in response to fenofibrate exposure. **(A)** Heatmap of significantly perturbed genes in the rat kidney (left panel) and liver (right panel) and the genes common between them (center panel) in response to increased fenofibrate doses. The total number of significantly perturbed genes (indicated in parentheses) for each tissue was obtained based on a false discovery rate <0.1 and log2 fold-change (FC) values > |0.3|. **(B,C)** Significantly perturbed biological pathways in the kidney and liver at the highest concentration based on KEGG **(B)** as well as Reactome and WIKI **(C)** pathway enrichment analysis. AFC, aggregated fold change.

Consistent with the transcriptomic profiles of the previous chemicals coumarin and PFOA, fenofibrate, a well-known PPARα agonist, induced a robust, dose-dependent transcriptional disruption that intensified at concentrations ≥125 mg/kg (Figure 6). Visualized via the manual clustering-based heatmap, we found that the liver mounted a substantially more expansive transcriptomic response than the kidney, yielding 703 unique DEGs (Figure 6A, right panel) compared to 470 in the kidney (Figure 6A, left panel), with 308 shared DEGs (Figure 6A, center panel). Specifically, several genes were significantly altered at the lowest dose, with the majority upregulated (red) in the liver compared to the kidney. The expression patterns for the shared genes indicated both commonalities (Custers 1, 3, and 4) and differences (Cluster 2) between the liver and kidney in response to fenofibrate exposure.

Pathway enrichment analyses of the altered genes indicated several highly upregulated gene clusters (indicated by red in Figure 6A), reflecting aggressive lipid metabolic reprogramming and adaptation, which confirmed fenofibrate’s potent mechanism of action, i.e., massive, shared fold enrichments in the PPAR signaling pathway, peroxisome activity, and fatty acid elongation across both organs (Figures 6B,C). However, Reactome profiling exposed a striking tissue-specific divergence: while the kidney exhibited a subdued stress response, the liver demonstrated a singular, massive spike in the detoxification of the ROS pathway (Figure 6C), indicating a severe redox imbalance and oxidative stress driven by excessive peroxisomal and mitochondrial fatty acid oxidation at high doses. Conversely, the downregulated gene clusters (indicated by green in Figure 6A, representing the suppression of baseline cellular homeostasis) mapped primarily to suppressed immune and signaling functions in both organs, including complement and coagulation cascades, focal adhesion, and some of the pathways related to carbohydrate metabolism. In addition, we also observed that the linoleic acid metabolism, steroid hormone biosynthesis, and ECM-receptor interaction pathways differed between the liver and kidney (Figure 6B, KEGG pathways). Furthermore, based on the Reactome and WIKI pathway analyses, we observed that several unique liver- and kidney-specific pathways were significantly enriched, indicating tissue specificity (Figure 6C). Collectively, these salient features demonstrate that while fenofibrate universally forced massive PPARα-mediated lipid reprogramming, it uniquely drove the liver toward extreme oxidative stress and potential toxicity at elevated exposures.

#### 3.4.1 Gene-level dose-response dynamics within key perturbed pathways following fenofibrate exposure

**FIGURE 7:**
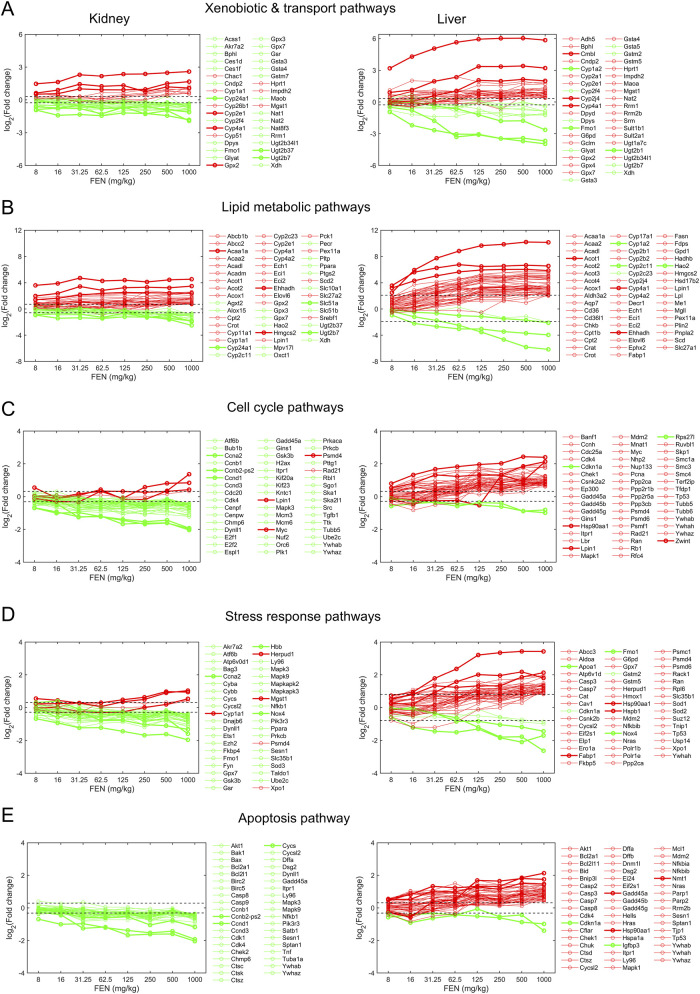
Pathway-based dose-response profiles for significantly perturbed genes in the kidney and liver in response to fenofibrate exposure. Line plots show the logarithmic fold-change (FC) values for genes significantly altered (false discovery rate <0.1) by fenofibrate in the **(A)** xenobiotic and transport pathways, **(B)** lipid metabolism, **(C)** cell cycle, **(D)** stress response, and **(E)** apoptosis pathways. Dashed lines show genes that satisfy the log2FC > |0.3| criterion at the highest dose of fenofibrate for all the figures except **(B,D)**. In **(B)**, the dashed line indicates the log2FC > |0.65| and > |2.0| for the left and right panels, respectively. In **(D)**, the dashed line indicates the log2FC > |0.8| for the right panel.

Figure 7 shows the individual dose-response trajectories of genes within the most significantly enriched biological pathways, illuminating how fenofibrate acts as a potent PPARα agonist to drive organ-specific molecular responses. Within the xenobiotic metabolism networks, we observed an aggressive adaptive response, characterized by the rapid, dose-dependent upregulation (indicated by red lines) of phase I clearance and functionalization genes, such as *Cyp4a1*, *Cyp2j4*, *Cyp51*, and *Cyp2e1*, in both the kidney (Figure 7A, left panel) and the liver (Figure 7A, right panel). However, in contrast, the phase II conjugation genes (several *Gpx*, *Gsta*, and *Ugt*) were significantly downregulated in the kidney compared to the liver.

This pronounced hepatic sensitivity extended deeply into the lipid metabolic pathways (Figure 7B). Fenofibrate triggered a massive transcriptional reprogramming in the liver, with several gene log2FC values > 2, drastically upregulating genes responsible for lipogenesis and peroxisomal fatty acid oxidation—most notably *Ehhadh*, *Acot1*, *Elovl6*, *Scd*, *Cpt1b*, *Cpt2*, *Hmgcs2*, and *Acaa1a* (Figure 7B, right panel). While the kidney did upregulate a smaller subset of lipid metabolism genes, critical drivers like *Cpt1b* and *Plin2* remained completely unchanged, reflecting a significantly lower magnitude of metabolic shift.

The gene expression profiles also captured strikingly divergent cellular growth trajectories between the two organs (Figure 7C). In the kidney, fenofibrate exposure forced a profound suppression of normal cellular division, evidenced by a widespread dose-dependent downregulation (indicated by green lines) of essential cell cycle drivers, kinases, and mitotic regulators, including *Ccnb1*, *Ccna2*, *Cdk4*, *Cdc20*, *Gadd45a*, and *Gins1*. Conversely, high-dose fenofibrate exposure pushed the liver to actively upregulate several of these proliferative markers (Figure 7C, right panel). This robust hepatic cell cycle activation strongly correlates with the known capacity of fibrate-induced PPARα activation to trigger pronounced hepatocellular hypertrophy, proliferation, and compensatory tissue regeneration in rodents (Xie et al., 2019; Yang et al., 2023).

However, this massive metabolic and proliferative hepatic overdrive comes at a severe cost. The excessive upregulation of peroxisomal and mitochondrial fatty acid oxidation in the liver generated dangerous levels of ROS, pushing the tissue into severe oxidative stress (Figure 7D). The liver uniquely attempted to counteract this redox imbalance by strongly upregulating key antioxidant defense genes, such as *Cat*, *Sod1*, and *Gpx7* (Figure 7D, right panel). Ultimately, as fenofibrate concentrations surpassed the 125-mg/kg transcriptomic inflection point, this compensatory antioxidant system became overwhelmed, triggering the activation of programmed cell death pathways (Figure 7E). The hepatic tissue actively upregulated critical mitochondrial cell death and apoptotic mediators, specifically *Bid*, *Cycsl2*, *Casp2*, *Casp3*, *Casp7*, and *Casp8*, marking the definitive onset of cellular degeneration and necrosis at peak exposure levels (Pannala et al., 2023). Throughout this high-dose stress event, the kidney remained largely unperturbed, maintaining baseline expression or downregulation across these exact same stress and apoptotic networks (Figure 7E, left panel).

### 3.5 Genome-scale metabolic modeling predicts chemical-specific fluxome alterations

To overcome the limitations of standard pathway-enrichment analyses, which do not account for the interaction of multiple genes or the complex connectivity between reaction networks in cellular metabolism, we integrated our HTT data with an updated rat GSM (Pannala et al., 2025b). We applied the Pheflux algorithm to predict the steady-state reaction rates—collectively known as the fluxome—across all metabolic subsystems for each rat (Hari et al., 2025). This genome-scale modeling approach allowed us to quantitatively predict how coumarin, PFOA, and fenofibrate fundamentally rewire the functional metabolic architecture of the liver and kidney.

**FIGURE 8:**
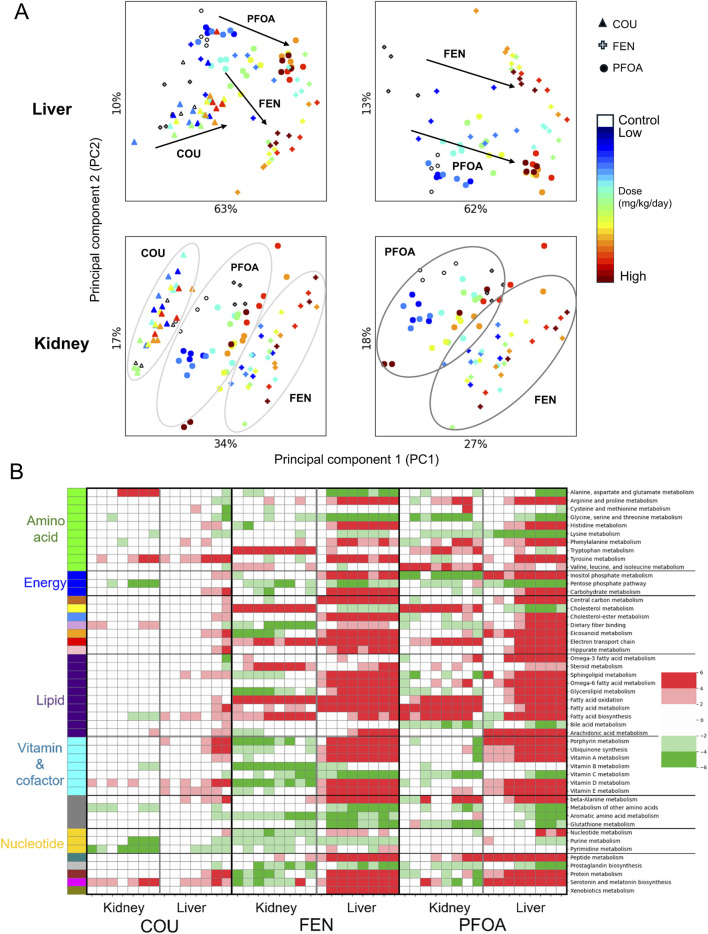
Genome-scale model-based predictions of metabolic perturbations in the liver and kidney. **(A)** Principal component-based analysis of metabolic subsystem-level flux activity compared for all three chemicals (left panels) and for fenofibrate and PFOA (right panels) in the liver (top panels) and kidney (bottom panels). **(B)** Comparison of the most significantly altered metabolic subsystems in the kidney and liver for each chemical. Each individual cell represents the z-score for the average metabolic subsystem fluxes per dose group with respect to the controls. Scores indicating significantly up- and downregulated pathway activity are shown in red and green, respectively. COU, coumarin; FEN, fenofibrate; PFOA, perfluorooctanoic acid.

Figure 8 shows the global and subsystem-level metabolic flux predictions, illustrating the profound biological shifts induced by the three chemicals. Figure 8A displays the PCA of the predicted metabolic subsystem fluxes. The PCA captured striking, dose-dependent metabolic trajectories that move distinctly outward from the control and low-dose groups (indicated by cool colors) to the high-dose groups (indicated by warm colors). In the liver, coumarin, PFOA, and fenofibrate drove massive and distinctly diverging metabolic shifts, confirming that each chemical induced a unique, high-magnitude functional reprogramming of the hepatic fluxome (Figure 8A, top panels). While the kidney also exhibited a clear dose-dependent clustering, the overall magnitude of separation from the controls was less expansive than in the liver, reinforcing our previous transcriptomic findings of pronounced hepatic sensitivity (Figure 8A, bottom panels).

Figure 8B shows the relative flux activity (z-score) in the metabolic subsystems as a heatmap, comparing kidney and liver exposure to the three chemicals with their respective controls. The analysis revealed that fenofibrate and PFOA—both acting as potent PPARα agonists—induced a massive and highly conserved lipid and energy metabolic overdrive, predominantly in the liver. This hyperactive state included strong flux increases in fatty acid oxidation, fatty acid biosynthesis, as well as fatty acid, sphingolipid, and eicosanoid metabolism. However, specific functional divergences exist: PFOA drove a strong upregulation of omega-3 fatty acid metabolism in the liver, whereas coumarin strongly suppressed it. To power this massive lipid processing, fenofibrate and PFOA concurrently triggered an energy overdrive in the liver, strongly upregulating electron transport chain, inositol phosphate, carbohydrate, and central carbon metabolism. Importantly, the heatmap exposes a highly chemical-specific disruption in sterol processing. Fenofibrate and PFOA strongly suppressed cholesterol and bile acid metabolism in the liver, while simultaneously upregulating cholesterol-ester metabolism. Because hepatic cholesterol homeostasis strictly requires the conversion of cholesterol to bile acids, this severe suppression directly drove cholesterol accumulation, hepatomegaly, and the progression of steatosis. In contrast, coumarin completely lacked this sterol-disrupting signature, instead mildly upregulating both cholesterol and bile acid metabolism at the highest doses.

The metabolic modeling further revealed complex, highly specific reorganization within amino acid networks. While broad categories like global “metabolism of amino acids” and glutathione metabolism were strongly suppressed across both organs for all the chemicals, specific amino acid pathways exhibited targeted shunting. Fenofibrate and PFOA strongly upregulated arginine, proline, and histidine metabolism in the liver, while strongly suppressing glycine, serine, threonine, and lysine metabolism. Notably, phenylalanine metabolism was strongly upregulated by PFOA (and mildly by fenofibrate) in the liver. However, a highly specific and universally conserved stress response emerged within tyrosine metabolism, which was strongly upregulated in the liver across all three chemicals (fenofibrate, PFOA, and coumarin). Meanwhile, branched-chain amino acid metabolism exhibited distinct tissue-specific partitioning, showing mild upregulation in the liver but strong upregulation in the kidney in response to PFOA. Finally, fenofibrate and PFOA exposure forced a massive, systemic upregulation of most vitamin and cofactor metabolic pathways, in addition to beta-alanine, protein, and serotonin/melatonin biosynthesis in the liver, while conversely suppressing these exact same vitamin pathways in the kidney.

Collectively, this genome-scale metabolic modeling provided a highly comprehensive and accurate picture of toxicity: while coumarin exhibited a distinct metabolic profile characterized by central carbon upregulation and omega-3 suppression, the PPARα agonists fenofibrate and PFOA forced the liver into a highly specific, hypertrophic metabolic state. This state aggressively prioritized massive lipid catabolism, energy production, and the shunting of specific amino acids (glycine and lysine) at the direct expense of bile acid homeostasis, cholesterol clearance, and glutathione metabolism, creating a profound functional imbalance that induces progressive tissue injury.

## 4 Discussion

**FIGURE 9:**
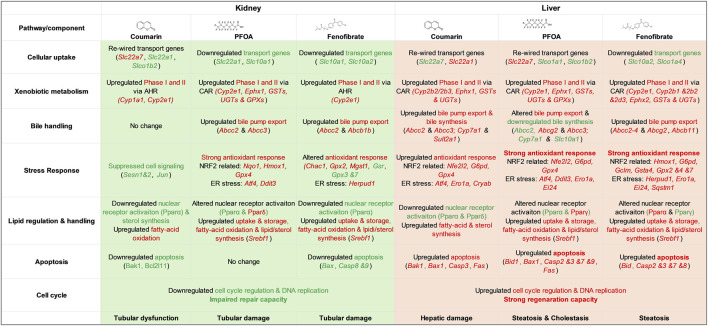
Summary of chemical-induced perturbations in liver and kidney metabolism for all three chemicals. Pathways and genes with significant up- and downregulation are shown in red and green, respectively. PFOA, perfluorooctanoic acid.

In this study, we aimed to delineate the early, organ-specific molecular events that precede overt toxicological injury by integrating short-term *in vivo* exposure models with advanced systems biology approaches (Gwinn et al., 2020; Hari et al., 2025). Specifically, we combined 5-day HTT data from rat liver and kidney tissues with GSM flux modeling to quantitatively predict how different chemical stressors fundamentally rewire cellular transcriptional responses and network-based metabolic flux distributions. To understand the mechanisms of the liver and kidneys’ responses to chemical exposures, we selected three distinct, widely studied chemicals: coumarin, fenofibrate, and PFOA. The fundamental rationale for comparing these specific chemicals lies in their shared molecular targets but divergent toxicological outcomes. All three chemicals are recognized in the scientific literature as agonists of PPARα, a master regulator of lipid homeostasis (Vanden Heuvel et al., 2006; Wang, 2010; Silva et al., 2024), but they represent distinctly different classes of compounds—a naturally occurring plant derivative (coumarin), a targeted synthetic pharmaceutical (fenofibrate), and a pervasive, multi-receptor-disrupting environmental toxicant (PFOA). Indeed, when we compared the significantly altered genes (FDR <0.1) at the highest dose across the three chemicals for each organ independently, we found that the genes that were common for the kidney or for the liver were identical, indicating some common mechanisms of action (Supplementary Table S1). These results suggest that gene perturbations that are specific to each chemical play a stronger role in their phenotypic outcome. While fenofibrate acts as an extraordinarily potent PPARα agonist, followed by PFOA (Evans et al., 2022), forcing the liver into a persistent, hypertrophic state of massive lipid and energy overdrive (Silva et al., 2024; Pannala et al., 2025a), coumarin represents a natural compound, known to indirectly activate PPARα, whose acute, high-dose toxicity is dominated by severe oxidative stress, generic tissue necrosis, and rapid cell-cycle regeneration (Lake et al., 1989; Niu et al., 2017; Liu et al., 2022). By comparing these three PPARα agonists of varying strengths, regulatory profiles, and application classes, this integrated transcriptomic approach effectively dissects how shared initial receptor targets can ultimately diverge into entirely distinct metabolic bottlenecks, cellular repair mechanisms, and apical organ injury outcomes. Figure 9 provides a high-level summary of the various cellular processes perturbed when the liver and kidneys are exposed to these studied chemicals.

### 4.1 Altered cellular uptake, xenobiotic metabolism, and bile acid handling

Across all three evaluated chemicals, the initial cellular interaction involved a significant disruption of transport mechanisms. We observed alterations in critical transport genes, such as *Slc22a7*, *Slc22a1*, and various *Slco* and *Slc10a* family members, in both the liver and kidney in response to all three chemicals (Figure 9). This disruption reflects the organs’ response to the pronounced partitioning and bioaccumulation of these chemicals, particularly PFOA and fenofibrate, which heavily utilize liver fatty acid-binding proteins and serum albumin for targeted cellular transport (Martin et al., 2013; Perez et al., 2013; Golden-Mason et al., 2025). Once the chemicals entered the organs, both the liver and kidney mounted several conserved xenobiotic defenses to clear the toxic insults by significantly upregulating phase I and phase II clearance enzymes (e.g., *Cyp2e1*, *Ephx1*, and various *Gsta* and *Ugt* family members). While coumarin predominantly upregulated several *Gsta* and *Ugt* family members in both the liver and kidney, we observed a mixed response to these enzymes for the other two chemicals, indicating impaired phase II conjugation mechanisms, particularly in the kidney (Figures 3, 5, 7). While fenofibrate and PFOA primarily engaged receptors like the constitutive androstane receptor (CAR) and pregnane X receptor (PXR) to drive this clearance (Attema et al., 2022), coumarin relied on the aryl hydrocarbon receptor (AHR) to induce classical carcinogen-detoxifying genes, such as Gsts and NAD(P)H quinone oxidoreductase (NQO1) (Itoh et al., 1997; Prince et al., 2009). Strengthening these observations at the gene level, our GSMs, which explored a combined effect of all these alterations at the system level, revealed significantly increased xenobiotic metabolism activity for fenofibrate exposure only in the liver, compared to the other two chemicals (Figure 8B), indicating the usefulness of system-level integration of multiple activated genes driving the overall fluxome.

Despite these shared early defense mechanisms, the three chemicals greatly differed in their subsequent handling of toxic metabolites and bile acids, especially within the liver (Figure 9, bile handling). Fenofibrate and coumarin upregulated bile pump export (*Abcc2*) and either maintained or enhanced bile synthesis (*Cyp7a1*), whereas PFOA uniquely forced the liver into a state of altered bile handling by upregulating export while strictly downregulating *de novo* bile acid synthesis. This severe suppression is driven by PFOA’s profound inhibition of cholesterol 7a-hydroxylase (*Cyp7a1*), the critical rate-limiting enzyme required for primary bile acid synthesis (Hari et al., 2024; Ma et al., 2024). Because hepatic cholesterol clearance strictly requires its conversion to bile acids, this PFOA-induced metabolic bottleneck disrupted enterohepatic circulation leading to progressive cholesterol accumulation. This altered bile acid handling may contribute to cholestasis development in the liver (Mariotti et al., 2018; Ma et al., 2024), a mechanism of injury that is entirely absent in the kidney due to its inherent lack of primary bile acid synthesis pathways and divergent lipid-handling capabilities.

### 4.2 Differential modulation of PPARα-mediated lipid regulation in liver and kidney metabolism

Our comparative analysis revealed that regulation of lipid metabolic pathways sharply differentiated the effects of coumarin from the other two PPAR agonists, i.e., PFOA and fenofibrate. The combination of dose-response profiles with GSM flux modeling revealed that while acute coumarin exposure downregulated the PPARα and PPARδ genes in both organs, it induced an uncoordinated lipid response characterized by upregulated generic fatty acid and sterol synthesis (Figures 3, 9) and suppressed omega-3 fatty acid metabolism in the liver as well as an isolated mild suppression of fatty acid biosynthesis in the kidney (Figure 8B). Conversely, PFOA and fenofibrate acted as extraordinarily potent agonists that fundamentally altered both PPARα and PPARγ activation in the liver with opposing feedback regulatory mechanisms (e.g., PPARα gene upregulated by fenofibrate but downregulated by PFOA), driving a massive PPAR-mediated transcriptomic upregulation of lipid uptake and storage (Figure 9) (Rakhshandehroo et al., 2010; Bougarne et al., 2018; de la Rosa Rodriguez et al., 2018; Golden-Mason et al., 2025). Concurrently, this receptor engagement forced the hepatic fluxome into an extreme lipid overdrive, marked by strongly increased steady-state metabolic fluxes through fatty acid oxidation, Srebf1-driven fatty acid biosynthesis, and eicosanoid, sphingolipid, and arachidonic acid metabolism (Figure 8B). Importantly, strengthening our observations from dose-response profiles, metabolic modeling revealed a severe, chemical-specific functional bottleneck in the liver: fenofibrate and PFOA powerfully suppressed cholesterol and primary bile acid metabolism while simultaneously upregulating cholesterol-ester metabolism, severely impairing hepatic cholesterol clearance and enterohepatic circulation (Post et al., 2001; Zhang et al., 2017; Hari et al., 2024).

In the kidney, both fenofibrate and PFOA downregulated the PPARα gene, but PFOA distinctly upregulated the PPARγ gene. Both chemicals significantly increased renal fluxes for fatty acid oxidation and uniquely upregulated renal cholesterol metabolism (Figure 8B), actively avoiding the sterol clearance bottleneck seen in the liver (Lee et al., 2024). Biologically, these divergent lipid-handling trajectories dictated distinct cellular consequences for each organ. For example, in the liver, exposure to fenofibrate and PFOA led to extreme PPAR-mediated lipid influx, unyielding fatty acid oxidation, and completely suppressed bile acid synthesis that overloaded the metabolic machinery, promoting lipotoxicity, which ultimately manifested as severe cellular hypertrophy, evidenced by increased absolute liver weights (Figure 1), cholestasis, and steatosis. Coumarin’s unique lipid profile led to generic hepatic tissue necrosis instead of a hypertrophic response, which is also evidenced by the unchanged liver and kidney weights. In contrast, the differential lipid handling disruptions in the kidney may culminate universally in sustained renal tubular damage, completely lacking the massive steatotic lipid accumulation and cholesterol-ester buildup characteristic of the hepatic response. Furthermore, some of these observed differences in lipid metabolism between the liver and kidney could also be influenced by the differences in basal expression of PPARα gene between the liver and kidney. The absolute gene expression counts from our control rats indicated a four times higher basal expression of PPARα gene in the kidney compared to the liver.

### 4.3 Alterations in antioxidant defense and integrated stress response pathways

Chemical exposures triggered pronounced cellular stress responses to counteract severe redox imbalances and metabolic toxicity. In the liver, PFOA, fenofibrate, and coumarin universally elicited massive activation of the Nrf2-mediated antioxidant defense system (including the upregulation of *Nfe2l2*, *G6pd*, *Gpx4*, and *Hmox1*) along with severe ER stress, evidenced by the induction of integrated stress response (ISR) and unfolded protein response (UPR) markers, such as *Atf4*, *Ddit3* (*Chop*), and *Ero1a* (Figures 3, 5, 7, 9). While Nrf2 activation initially served as a critical cytoprotective mechanism to neutralize overwhelming ROS and prevent acute tissue damage, the sustained upregulation of the ISR actively drove the progression of apical liver injury. The literature confirms that prolonged ER stress stimulates *Chop* gene induction, forcing the UPR to switch from an adaptive homeostatic response to a pathogenic driver of programmed cell death (Hocevar et al., 2020). Correspondingly, our transcriptomic analysis demonstrated that the liver aggressively upregulated apoptotic execution pathways in response to all three chemicals to clear damaged cells. Coumarin induced pro-apoptotic markers, such as *Bak1*, *Bax*, *Casp3*, and *Fas* (Figures 3, 9), driving classical hepatic tissue turnover. Similarly, the massive lipid overdrive induced by PFOA and fenofibrate forced the liver into a persistent state of lipotoxicity, triggering widespread upregulation of intrinsic and extrinsic apoptotic mediators, including Bid, Bax, Fas, and multiple executioner caspases (*Casp2*, *Casp3*, *Casp7*, *Casp8*, *Casp9*) (Figures 5, 7, 9). These results align with established models showing that PFOA induces apoptosis in hepatic cells through mitochondrial and ER-stress-dependent pathways (Qi et al., 2023). This active apoptotic clearance, paired with robust cell cycle progression, facilitated a strong regenerative capacity in the liver, which was manifested as progressive cellular hypertrophy and steatosis rather than immediate necrotic failure.

In stark contrast, the kidney exhibited highly divergent stress handling and apoptotic strategies that fundamentally altered its injury trajectory. While the liver utilized robust ISR and Nrf2 activation to drive aggressive apoptotic tissue turnover, the kidney mounted a restricted or entirely suppressed response. During coumarin exposure, the kidney paradoxically repressed baseline cellular stress signaling (downregulating *Sesn1*, *Sesn2*, and *Jun*) rather than mounting an ISR or antioxidant defense (Figures 3, 9). Although PFOA and fenofibrate maintained a coordinated ER stress (*Atf4*, *Herpud1*) and antioxidant response in the renal tissue, they actively evaded or failed to induce the subsequent apoptotic execution pathways. Specifically, fenofibrate and coumarin strictly downregulated pro-apoptotic drivers (*Bak1*, *Bcl2l11*, *Bax*, *Casp8*, *Casp9*) in the kidney, while PFOA merely maintained apoptotic signaling at baseline levels (Figures 3, 5, 7, 9). Consequently, because it actively suppressed programmed cell death and lacked the robust, ISR-driven apoptotic clearance characteristic of the hepatic response, the kidney had a profoundly impaired repair capacity, which could ultimately be manifest as stagnant, irreversible tubular damage.

### 4.4 Chemical exposure induced divergent cell cycle regulation fates in liver and kidney metabolism

Chemical exposure induced highly divergent cell cycle regulation and DNA replication responses between the liver and kidney, ultimately dictating the distinct regenerative capacity of each organ. In the liver, coumarin, PFOA, and fenofibrate universally upregulated cell cycle regulators, mitotic kinases, and DNA replication machinery, including *Cdk1*, *Ccnb1*, and *Mcm* complex genes (Figures 3, 5, 7, 9). The liver actively coupled this robust proliferative response with pronounced apoptotic execution to efficiently clear damaged cells and counteract the severe oxidative and ER stress generated by either generic tissue necrosis (coumarin) or a massive receptor-mediated lipid overdrive (PFOA and fenofibrate). This compensatory regeneration aligns closely with established *in vivo* models demonstrating that potent PPARα agonists, such as fenofibrate and PFOA, directly stimulated hepatocyte proliferation, hepatomegaly, and active tissue turnover to manage extreme lipid accumulation and lipotoxicity (Yamamoto et al., 1996; Qi et al., 2023; Golden-Mason et al., 2025). Literature evidence suggests that under long-term administration, the activation of PPARα is found to be hepatocarcinogenic in rodents, a mechanism related to the downregulation of let-7c micro-RNA expression, which stabilizes Myc mRNA, contributing to increased mitogenic signaling and the consequent hepatocyte proliferation. This is an effect via both PPARα-dependent and -independent pathway, which has been testified to be absent in humans (Peters et al., 2012). In stark contrast to the hepatic response, the kidney strictly downregulated essential cell cycle drivers and DNA replication pathways in response to all three chemical exposures (Figures 3, 5, 7, 9). Because the kidney failed to mount a robust apoptotic response to efficiently clear cells damaged by the chemical insults, oxidative stress, and localized lipid dysregulation, this profound cell cycle repression trapped the renal tissue in a stagnant cellular state. Consequently, the kidney completely lacked the dynamic, compensatory tissue regeneration observed in the liver, causing an impaired repair capacity, which directly translated the initial metabolic and stress-induced injuries into sustained, irreversible tubular damage if chemical exposure persisted. Furthermore, differences in the tissue distribution of these chemicals between the liver and kidney (Piller, 1977; Chapman, 1987; Kudo et al., 2007) may also contribute to the observed stronger perturbations in the liver, given its primary role in metabolism.

## 5 Limitations

In this study, we exclusively used experimental data from only male Sprague-Daley rats since the original study did not include female rat data for comparison. Therefore, the organ-level observations made in this study may not be generalizable to female rats due to inherent sex-level differences in metabolism between male and female rats. Furthermore, we used all the datasets from a targeted TempO-seq platform that measured only ∼3,000 transcripts, which indicates that a large fraction of metabolic genes were not covered for the rat GSM integration. However, we tested the impact of this low-coverage issue by using the previously published extrapolated datasets that contained a much larger coverage (75%) for the same chemicals (Pannala et al., 2025b), and the results indicated that our analysis using the S1500+ dataset was able capture several core pathways, confirming the ability of the platform to predict overall organ metabolism. Although our integrated rat GSM analysis was able to identify major pathway perturbations robustly, we would require the corresponding experimental flux distributions measured for the same chemicals under similar conditions for validation. Therefore, these rat GSM results should be used to generate hypotheses for future experimental studies.

## 6 Conclusion

In this study, we integrated 5-day *in vivo* high-throughput transcriptomic data with genome-scale metabolic modeling to successfully predict early, organ-specific mechanisms of chemical toxicity. By evaluating three functionally distinct PPARα agonists using this computational framework, we effectively distinguished between the generic, necrosis-driven tissue damage and paradoxical PPAR suppression due to acute coumarin exposure and the massive, receptor-mediated lipid overdrive induced by fenofibrate and PFOA. In the highly sensitive liver, fenofibrate and PFOA aggressively upregulated lipid uptake, unyielding fatty acid oxidation, and profound ER stress, which actively drove lipotoxicity, progressive cellular hypertrophy, and steatosis. Furthermore, we found that the liver and kidney exhibited fundamentally divergent stress-handling and repair trajectories. While the liver mounted robust apoptotic execution and cell-cycle regenerative responses to counteract severe chemical insults, the kidney actively repressed these vital repair pathways, thereby causing a profoundly impaired regenerative capacity, which translated metabolic damage into irreversible tubular dysfunction. Ultimately, our combined genome-scale and pathway-based transcriptomic analysis identified the precise metabolic bottlenecks, such as PFOA’s suppression of bile acid synthesis, and the divergent cellular survival strategies that dictate distinct apical organ injury outcomes, providing a highly sensitive, predictive framework for next-generation chemical risk assessment. This study’s relevance to human health lies in its demonstration that integrating short-term transcriptomic responses with genome-scale metabolic modeling can identify early, organ-specific mechanisms of toxicity and distinguish between fundamentally different modes of chemical action before overt injury occurs. By differentiating acute necrosis-driven toxicity and metabolic suppression induced by coumarin from the receptor-mediated lipid metabolic overload caused by fenofibrate and PFOA, this approach provides mechanistic insight into how environmental contaminants and pharmaceutical agents may contribute to liver and kidney dysfunction in humans.

## Data Availability

The original contributions presented in the study are publicly available. This data can be found in the NCBI GEO repository with the accession number GSM4415261 at https://www.ncbi.nlm.nih.gov/geo/query/acc.cgi?acc=GSM4415261.
